# Association between DNA Methyltransferases 3B Gene Polymorphisms and the Susceptibility to Acute Myeloid Leukemia in Chinese Han Population

**DOI:** 10.1371/journal.pone.0074626

**Published:** 2013-09-17

**Authors:** Qin Zheng, Ting-ting Zeng, Jiao Chen, Hua Liu, He Zhang, Jun Su

**Affiliations:** Department of Laboratory Medicine, West China Hospital of Sichuan University, Chengdu, China; Gentofte University Hospital, Denmark

## Abstract

DNMT3B plays a crucial role in the generation of aberrant methylation during carcinogenesis. Polymorphisms in the DNMT3B gene may influence the DNA methylation enzymatic activity of DNMT3B, thereby modulating the susceptibility to AML. Thus, we investigated the association between SNPs in the DNMT3Bgene and their haplotypes with the risk of AML in the Chinese Han population. The DNMT3B genotype was determined by HRM in 317 de novo AML patients and 406 healthy control subjects matched for age and gender. Among the 5 SNPs investigated in this study, rs2424913 demonstrated no polymorphisms in the Chinese Han populations, rs1569686 and rs2424908 were significantly associated with AML risk. The GG genotype of rs1569686 was associated with increased AML risk (OR: 5.76; 95%CI: 2.60-12.73; *P*<0.01) compared with the TT genotype, and individuals with a G allele had a significantly increased risk (OR: 1.89; 95%CI: 1.41-2.52; *P<0.01*) for AML compared with those harboring a C allele, this polymorphism can predict the risk of AML in a minority of patients. While the CC genotype of rs2424908 appeared to reduce the AML risk (OR: 0.57; 95%CI: 0.36-0.91; *P*=0.01) compared with the TT genotype, individuals with a C allele were associated with a lower risk (OR: 0.79, 95%CI: 0.64-0.97, *P*=0.03) for developing AML compared with those harboring a T allele. The other 2 SNPs, rs6087990 and rs6119954, had no significant association with AML risk in the study population. The CGGT, CTAT, TGAT, and CGAT haplotypes of rs6087990, rs1569686, rs6119954, and rs2424908 appeared to significantly increase the AML risk, and the TTGC haplotype appeared to significantly reduce the risk. These results suggest that DNMT3B polymorphisms may contribute to the genetic susceptibility to AML; in particular, the G allele of rs1569686 serves as a risk factor for AML, whereas the C allele of rs2424908 represents a potential protective factor.

## Introduction

Acute myeloid leukemia (AML) is the most common acute leukemia in adults and is a heterogeneous clonal disorder characterized by the uncontrolled proliferation of neoplastic hematopoietic precursor cells that lose the ability to differentiate into mature cells. This loss of mature cells leads to fatal infection, bleeding, or organ infiltration within 1 year of diagnosis in the absence of treatment [1].

In the past few years, studies have shown that risk factors for acquiring AML include exposure to ionizing radiation, benzene, and cytotoxic chemotherapy [2-4]. Currently, it is generally accepted that both environmental and genetic factors are involved in the pathogenesis of AML. In particular, interactions between genetic and epigenetic changes may participate in the pathogenetic mechanisms of this disorder [5-9]. Furthermore, knowledge of the pathophysiology of AML has led to the development of novel treatment strategies, which, unlike traditional cytotoxic therapies, use epigenetic agents to modulate gene expression.

Epigenetic gene regulation results from the chemical modification of DNA and histones, e.g., DNA methylation and histone acetylation. Among the different epigenetic changes, DNA methylation plays an important role in the pathogenesis of hematological malignancies by silencing tumor suppressor genes and enhancing the expression of genes involved in cell proliferation and differentiation [10-12].

In mammals, DNA methylation consists of the covalent post-replicative addition of a methyl group to carbon 5 of the cytosine in a CpG dinucleotide, which is catalyzed by DNA methyltransferases (DNMTs) [13]. DNMTs, the key enzymes for genome methylation, include DNMT1, DNMT2, DNMT3A, DNMT3B, and DNMT3L, and each plays a different functional role [14,15]. DNMT1 primarily acts as a maintenance methyltransferase during each cell division and copies DNA methylation patterns to daughter strands during DNA replication. DNMT2, which is also called TRDMT1 (transfer RNA aspartic acid methyltransferase), possesses enigmatic biological functions, is distinct and highly conserved among taxa, and participates in both DNA and RNA methylation [16]. DNMT3A and DNMT3B are considered to be de novo DNA methyltransferases that establish DNA methylation patterns early in development. DNMT3L methylation is thought to stimulate the de novo methylation machinery by interacting with the catalytic domains of DNMT3A and DNMT3B, but DNMT3L has no catalytic activity [17]. These DNMTs are believed to establish and maintain DNA methylation patterns, and aberrant DNA methylation and DNMT gene polymorphisms have been detected in several cancers [18,19], although the precise mechanisms underlying this link remain elusive.

The DNMT3B gene is located on chromosome 20q11. 2 and encodes DNA methyltransferase 3b (DNMT3B), the protein required for genome-wide de novo methylation, the establishment of DNA methylation patterns during development, and the regulation of the histone code and DNA methylation at centromeric regions. DNMT3B promoter polymorphisms have been reported to be associated with the risk of immune thrombocytopenic purpura (ITP) and malignant solid tumors, such as colorectal cancer, lung cancer, and breast cancer, albeit to varying degrees [18-22]. However, there is little information regarding the role of DNMT3B polymorphisms in the development of hematologic malignancies.

It is well known that methylation of promoter and exon regions is one of the major regulatory mechanisms of gene expression; thus, we hypothesized that genetic variants of DNMT3B may be responsible for regulating the methylation status of other genes associated with AML risk. In this hospital-based, case-control study including 317 de novo AML patients and 406 healthy control subjects frequency-matched by age, sex, and ethnicity, we genotyped DNMT3B gene polymorphisms to evaluate the association between this genetic variant and susceptibility to AML.

## Materials and Methods

### Ethics statement

Written informed consent was obtained from all participants before enrollment in this study, and written informed consent for minor participants was obtained from their parents or legal guardian. This study was approved by the Ethics Committee of West China Hospital of Sichuan University.

### Study subjects

Between September 2010 and January 2013, 317 de novo AML patients without previous treatment were enrolled in this study in the Department of Hematology, West China Hospital of Sichuan University. Diagnosis was performed according to clinical, morphological, cytochemical, and immunophenotypic examination. A total of 406 healthy control subjects were recruited randomly from the West China Hospital of Sichuan University during the same period. We defined a healthy subject as a person who was free of disease (including no history of hematological diseasesor solidtumor) upon health check-up. The controls and cases were well matched for age and gender. All participants were ethnically of Chinese Han descent and resided in West China or its surrounding regions. A standardized questionnaire was used to collect basic health statistics information.

### DNA extraction

Mononuclear cells from peripheral blood samples or bone marrow from AML patients and mononuclear cells from peripheral blood samples from control subjects were isolated by Ficoll gradient centrifugation, aliquoted into fetal calf serum with 10% dimethyl sulfoxide (DMSO), and stored at -70°C until DNA extraction. Genomic DNA was prepared from mononuclear cells using the QIAamp® DNA Blood mini kit (Qiagen, Germany) according to the manufacturer’s protocol and diluted to 10 ng/µL for analysis. Extracted DNA samples were stored at -70°C.

### Single nucleotide polymorphism (SNP) selection and genotyping

In this study, 5 SNPs were selected from the promoter and exon regions of the DNMT3B geneusing the HapMap Project and Single Nucleotide Polymorphism database, and referenced sequences were taken from the dbSNP public database (http://www.ncbi.nlm.nih.gov/SNP/index.html). Primers were designed to amplify small fragments (approximately 55-110bp) covering the polymorphism loci. The selected SNPs and primer sequences are shown in Table 1. Polymorphisms of the 5 SNPs were detected using the high-resolution melting (HRM) method. Polymerase chain reaction (PCR) amplification and the HRM procedures for variants of these SNPs were performed in 96-well plates using the LightCycler® 480 Real-Time PCR System (Roche Diagnostics, Penzberg, Bavaria, Germany). A20μL PCR reaction mixture contained the following reagents: 1.0µL purified genomic DNA (10 ng/µL), 1.0µL forward primer, 1.0µL reverse primer (TAKARA, Japan), 1.0µL 20× EvaGreen™ Dye (Biotium, USA), 2.0µL dNTPs (2.5mM), 0.2µL Hot Star Taq® Plus DNA Polymerase (TAKARA, Japan), 2.0µL 10× buffer(TAKARA, Japan), 2.0µL 25mM MgCl_2_, and 9.8µl H_2_O.

**Table 1:** Primer sequences for genotyping DNMT3B gene polymorphisms.

**Reference SNP ID**	**Reference SNP Alleles**	**Primer sequences**
rs2424913	C/T	F:5' CTTCCAGTTGTCCTGAAGCTGGCTA 3'
		R:5' CTCACTGGGCCTTAGGTGACTGG 3'
rs6087990	T/C	F:5' CCGTTCGGGTTGAAAGGA 3'
		R:5' TGGATCAGAAGCCCTAAGCG 3'
rs1569686	G/T	F:5' GAACAAAAAGCAAAGGCAAGTGACT 3'
		R:5' GAACGAGTAAAAAACTTCAGGGCAT 3'
rs6119954	G/A	F:5' TGTTACAGTGGCAGTGTGGGATGAT 3'
		R:5' CCCATCACAAGCCAACTGCTTTATC 3'
rs2424908	C/T	F:5' ATTCTGCTCCAATGCTGCCCCTCAT 3'
		R:5' GCCCCAAGGAGTGGTCAATGGTAAC 3'

PCR cycling conditions consisted of an initial denaturation at 95°C for 15 min followed by 50 cycles of denaturation at 94°C for 10 s, annealing at 58°C (60°C for rs2424913 and rs2424908) for 15 s, and extension at 72°C for 25 s. After amplification, the PCR products were denatured at 95°C for 1 min and cooled to 40°C for 1 min to form double-stranded DNA.HRM analyses were then performed by gradually increasing the temperature from 65 to 95°C at a rate of 0.01 °C/s. After the melting procedure, the instrument was cooled to 40°C. The temperature data and melting curves were analyzed with the LightCycler® 480 Gene Scanning software v1.2 (Roche Diagnostics, Germany). Software programs employed a 3-step analysis consisting of (1) normalization by manually selecting linear regions before (100% fluorescence) and after (0% florescence) the melting procedure; (2) temperature shifting by selecting thresholds; and (3) automatic grouping by calculation. Different genotypes of the SNP positions were then distinguished according to normalized melting curves. The genotypes of the samples were defined based on reference samples with known genotypes.

In the experimental and control groups, at least 2-3 DNA samples for each genotype of each SNP had previously been genotyped by sequencing. Three samples with different genotypes for each SNP were added in each run of the HRM reactions as reference samples, and the results of the HRM reactions and sequencing were 100% concordant.

### DNA sequencing analysis

To confirm the genotyping results, PCR-amplified DNA samples were selected and examined by DNA sequencing. The sequencing primers were the same primers used in the PCRs.The PCR products were purified using the shrimp alkaline phosphatase method. The DNA sequences of the PCR products were determined with the BigDye Terminator v3.1 Cycle Sequencing Kit and ABI 3130 genetic analyzer (Applied Biosystems). The results obtained from the DNA sequencing analysis confirmed the reliability of the genotyping assay.

### Statistical analysis

The cases and controls were compared using the independent samples T test or Mann-Whitney U test for continuous variables and the Chi-square (χ^2^) test for categorical variables. Hardy-Weinberg equilibrium (HWE) was determined by the goodness-of-fit χ^2^ test with one degree of freedom to compare the observed genotype with the expected genotype frequencies for the subjects. Logistic regression models were used to examine whether the DNMT3B gene polymorphisms were associated with AML. Odds ratios (ORs) and 95% confidence intervals (95%CIs) were calculated using logistic regression analysis and the χ^2^ test. All of these data, in addition to linkage disequilibrium (LD) analysis, haplotype analysis and P values, were calculated. Statistical analyses were performed using SPSS 13.0 (SPSS, Chicago, IL, USA) and the Haploview 4.2 software (Daly Lab, Cambridge, MA, USA) with the online tool SNPstats (http://bioinfo.iconcologia.net/SNPstats). All statistical analyses were 2-tailed, and the significance level was set at 0.05.

## Results

**Figure 1:**
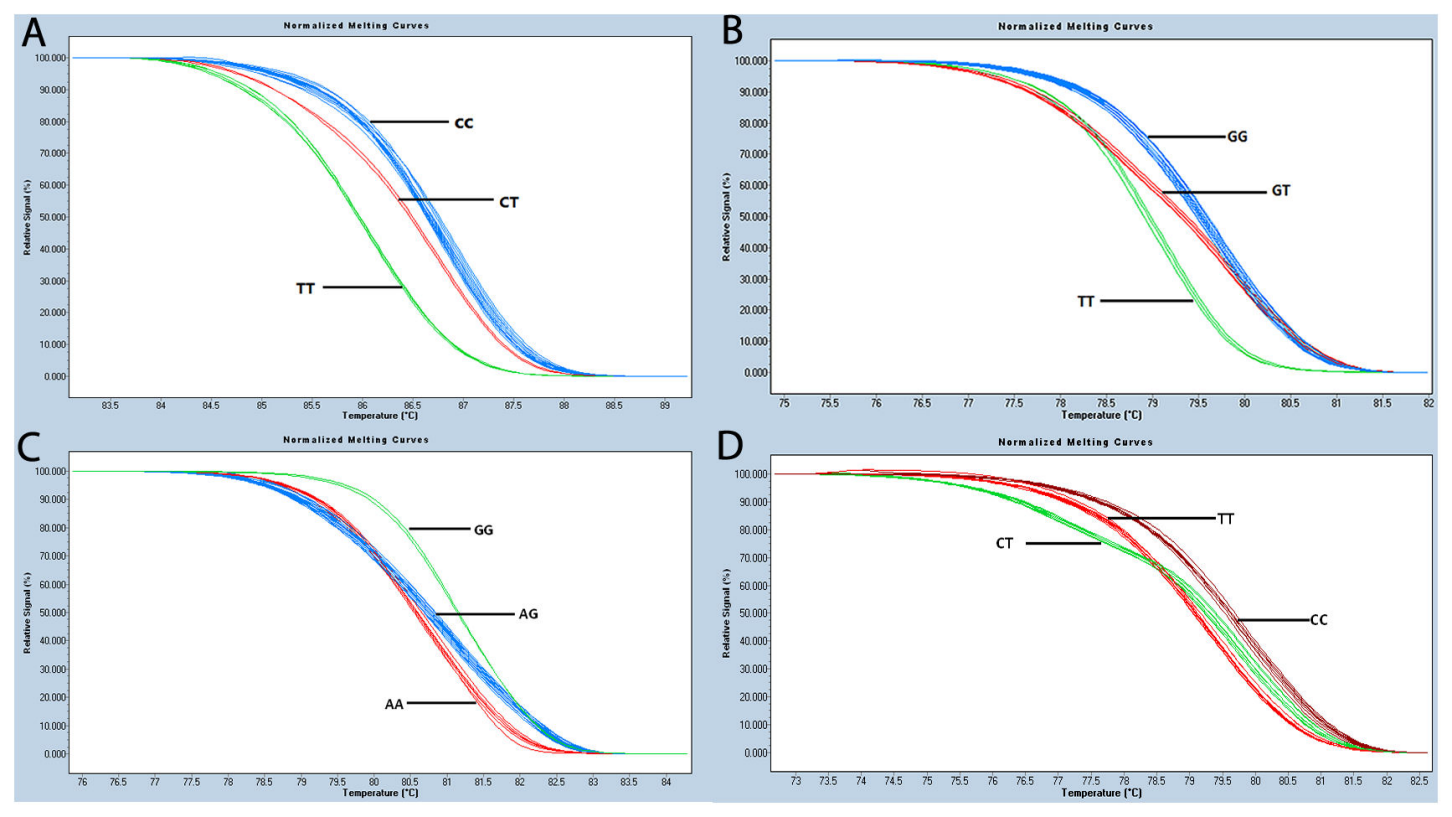
The melting curves of genotype of SNPs in DNMT3B gene. The three groups are well distinguished: A. rs6087990: TT in green, CC in blue and CT in red; B. rs1569686: TT in green, GG in blue and GT in red; C. rs6119954: AA in red, GG in green and AG in blue; D. rs2424908: TT in red, CC in brown and CT in green.

Among the 5 SNPs investigated in the DNMT3B gene in this study, rs2424913 (-149C>T) demonstrated no polymorphisms in either the case or control groups from the Chinese Han population (100% genotype TT). Therefore, this site was excluded from subsequent analyses. The remaining 4 SNPs, rs6087990, rs1569686, rs6119954, and rs2424908, were successfully genotyped, and melting curves for genotyping the SNPs using HRM were shown in Figure 1.

### Subject characteristics

A total of 317 de novo AML patients and 406 healthy controls were enrolled in this study, and the clinical characteristics of the participants are detailed in Table 2. Males accounted for 57.7% of the cases compared with 58.6% of the controls. The median age (at diagnosis) of the patients was 48 years (range: 14-81 years), and that of the controls was 47.5 years (range: 13-84 years). The gender and age distribution of the cases and controls indicated no statistically significant difference (*P*>0.05).

**Table 2:** Clinical characteristics of the AML patients group and healthy control group.

**Characteristic**		**Cases N (%**)	**Controls N (%**)	***P* value**
**Sample size**		**317**	**406**	-
**Sex**	Male	183 (57.7)	238 (58.6)	0.81^a^
	Female	134 (42.3)	168 (41.4)	-
**Age (years**)	Median	48	47.5	0.45^b^
	Range	14-81	13-84	-
**FAB classification**	M0	7 (2.2)	-	-
	M1	36 (11.4)	-	-
	M2	144 (45.4)	-	-
	M3	33 (10.4)	-	-
	M4	40 (12.6)	-	-
	M5	35 (11.0)	-	-
	M6	21 (6.6)	-	-
	M7	1 (0.4)	-	-
**Cytogenetic**	AML1/ET0(+)	14	-	-
**Abnormalities**	PML/RARα(+)	30	-	-
	CβFB/MYH11(+)	5	-	-
**White Blood Cell**	Median	7.44	5.80	0.02^c^
**Count (10^9^/L**)	Range	0.43-556.20	4.11-9.64	-
**Hemoglobin**	Median	74	143	<0.01^c^
**Level (g/L**)	Range	31-157	111-163	-
**Platelet Count**	Median	38	177	<0.01^c^
**(10^9^/L**)	Range	3-260	85-320	-

^a^ Chi-square (χ^2^) test; ^b^ Independent-Samples T test; ^c^ Mann-Whitney U test

### Association between individual SNPs in the DNMT3B gene and AML risk

The distribution of the genotypes and allele frequencies for the rs6087990, rs1569686, rs6119954, and rs2424908 SNPs in the DNMT3B gene in AML patients and control subjects is summarized in Tables 3 and 4. The distribution of the genotypes for all 4 SNPs in the control subjects was in agreement with HWE (*P* >0.05).

**Table 3:** Association of *DNMT3B* genotypes with the susceptibility to acute myeloid leukemia.

**SNP**	**Genotype**	**Case N (%**)	**Control N (%**)	**OR (95%CI**)*	*P* value*	**HWE *P* value** ^#^
rs6087990	CC	195 (61.5)	250 (61.6)	1.0(Ref)	-	0.16
	CT	98 (30.9)	114 (28.1)	1.10 (0.79-1.53)	0.56	-
	TT	24 (7.6)	42 (10.3)	0.73 (0.43-1.25)	0.26	-
rs1569686	TT	221 (69.7)	318 (78.3)	1.0(Ref)	-	0.27
	GT	64 (20.2)	80 (19.7)	1.15 (0.79-1.67)	0.46	-
	GG	32 (10.1)	8 (2.0)	**5.76 (2.60-12.73)**	**<0.01**	-
	GT+GG	96 (30.3)	88 (21.7)	**1.57 (1.12-2.20)**	**0.01**	-
rs6119954	GG	139 (43.8)	194 (47.8)	1.0(Ref)	-	0.15
	GA	155 (48.9)	182 (44.8)	1.19 (0.88-1.61)	0.27	-
	AA	23 (7.3)	30 (7.4)	1.07 (0.60-1.92)	0.82	-
rs2424908	TT	110 (34.7)	117 (28.8)	1.0(Ref)	-	0.12
	CT	168 (53.0)	216 (53.2)	0.83 (0.60-1.15)	0.26	-
	CC	39 (12.3)	73 (18.0)	**0.57 (0.36-0.91)**	**0.01**	-
	CT+CC	207 (65.3)	289 (71.2)	0.76 (0.56-1.05)	0.09	-

OR: Odds Ratio 95% CI: 95% confidence interval HWE: Hardy-Weinberg equilibrium Ref: reference

# Hardy-Weinberg equilibrium test for control * Calculated by binary logistic regression analysis

**Table 4:** Impact of the number of risk alleles on susceptibility to acute myeloid leukemia.

**SNP**	**Allele**	**Case N (%**)	**Control N (%**)	**OR (95%CI**)*	*P* value*	**Minor Allele**	MAF
rs6087990	C	488 (77.0)	614 (75.6)	1.0(Ref)	-	T	0.24
	T	146 (23.0)	198 (24.4)	0.93 (0.73-1.19)	0.55	-	-
rs1569686	T	506 (79.8)	716 (88.2)	1.0(Ref)	-	G	0.12
	G	128 (20.2)	96 (11.8)	**1.89 (1.41-2.52)**	**<0.01**	-	-
rs6119954	G	433 (68.3)	570 (70.2)	1.0(Ref)	-	A	0.30
	A	201 (31.7)	242 (29.8)	1.09 (0.87-1.37)	0.44	-	-
rs2424908	T	388 (61.2)	450 (55.4)	1.0(Ref)	-	C	0.45
	C	246 (38.8)	362 (44.6)	**0.79 (0.64-0.97)**	**0.03**	-	-

MAF: Minor allele frequency Ref: reference * Calculated by Chi-square (χ^2^) test

Notably, rs6087990 and rs6119954 demonstrated no significant differences in genotype distribution and allele frequency between patients with AML and control subjects.

In contrast, rs1569686 and rs2424908 appeared to be strongly associated with AML susceptibility. The genotype distribution in the AML patient group for the rs1569686 SNP was 69.7% TT, 20.2% GT, and 10.1% GG, which was significantly different from that of the control group (78.3% TT, 19.7% GT and 2.0% GG; *P<0.01*). Compared with the TT genotype, the heterozygous genotype GT showed no AML risk relationship, but the GG genotype appeared to increase the AML risk(OR: 5.76; 95%CI: 2.60-12.73; *P<0.01*) under an additive model, and the GT+GG genotype appeared to increase the AML risk(OR:1.57; 95%CI: 1.12-2.20;*P*=0.01) under a dominant model. Consistently, the frequency of the G allele appeared to be significantly increased for the rs1569686 SNP in the AML patient group (OR: 1.89; 95%CI: 1.41-2.52; *P<0.01*). For the rs2424908 SNP, compared with the TT genotype, the CC genotype appeared to reduce the AML risk (OR: 0.57; 95%CI: 0.36-0.91; *P*=0.01) in an additive model; however, the CT+CC genotype had no significant difference (OR: 0.76; 95%CI: 0.56-1.05; *P*=0.09) in a dominant model. Likewise, for rs2424908, the C allele was associated with a lower risk for developing AML (OR: 0.79; 95%CI: 0.64-0.97; *P*=0.03).

### LD and haplotype analysis

**Figure 2:**
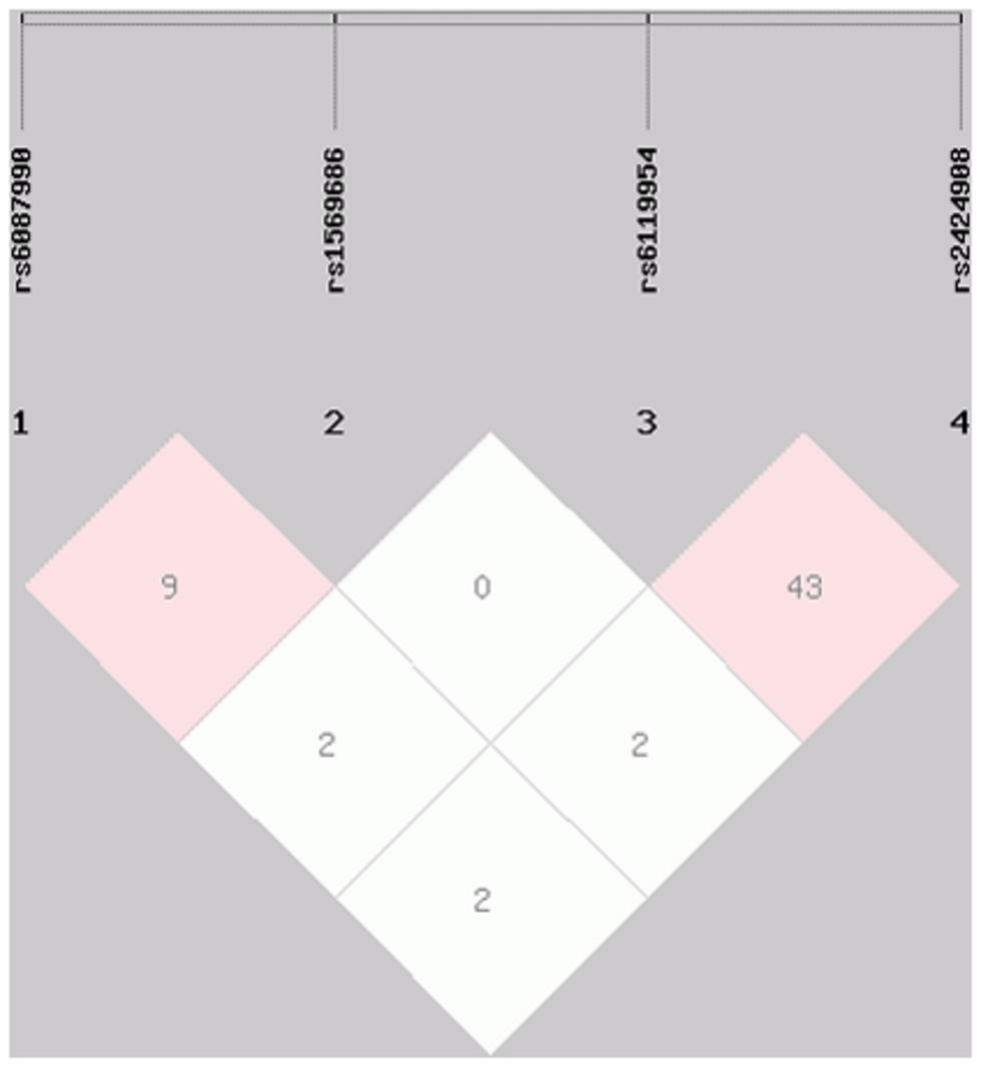
Linkage disequilibrium structure of the selected 4 SNPs on *DNMT3B* gene (rs6087990, rs1569686, rs6119954 and rs2424908) Values were showed in r^2^.

To evaluate the correlations of the 4 SNPs selected in the DNMT3B gene, we performed LD and haplotype analysis. The LD structure of the 4 SNPs was shown in Figure 2. The rs6087990, rs1569686, rs6119954, and rs2424908 SNPs in the DNMT3B gene exhibited weak LD (D'≤0.84; r^2^≤0.43). Haplotype analysis of the 4 SNPs was performed, and the 15 haplotype associations in the case and control groups are shown in Table 5. Compared with the major haplotype CTGT, the CGGT, CTAT, TGAT, and CGAT haplotypes appeared to be significantly increased in patients with AML compared to control individuals (OR: 5.17,95%CI: 2.52-10.59,*P<0*.*01*;OR: 8.38, 95%CI: 2.44-28.76, *P<0.01*; OR: 6.99, 95%CI: 1.52-32.15, *P*=0.01; OR: 2.34, 95%CI: 2.18-2.63, *P<0.01*, respectively). In contrast, the TTGC haplotype appeared to be significantly reduced in patients with AML compared to healthy subjects (OR: 0.29; 95%CI: 0.12-0.71; *P<0.01*).

**Table 5:** Results of haplotype analysis and its potential association with the susceptibility to AML.

**No.**	**rs6087990**	**rs1569686**	**rs6119954**	**rs2424908**	**Case (freq**)	**Control (freq**)	**OR (95%CI**)	*P* value*
1	C	T	G	T	252 (0.398)	352 (0.434)	1.0(Ref)	-
2	C	T	A	C	115 (0.182)	187 (0.230)	0.86 (0.65-1.14)	0.32
3	T	T	G	T	57 (0.090)	72 (0.088)	1.11 (0.75-1.62)	0.62
4	T	G	G	C	39 (0.061)	54 (0.067)	1.01 (0.65-1.57)	1.00
5	C	G	G	T	37 (0.059)	10 (0.012)	**5.17 (2.52-10.59)**	**<0.01**
6	C	T	G	C	30 (0.047)	42 (0.052)	0.99 (0.61-1.64)	1.00
7	T	T	A	C	28 (0.045)	28 (0.034)	1.40 (0.81-2.42)	0.26
8	C	G	A	C	22 (0.035)	16 (0.020)	1.92 (0.99-3.73)	0.06
9	T	T	G	C	6 (0.009)	29 (0.035)	**0.29 (0.12-0.71)**	**<0.01**
10	C	T	A	T	18 (0.028)	3 (0.004)	**8.38 (2.44-28.76)**	**<0.01**
11	T	G	A	T	10 (0.016)	2 (0.002)	**6.99 (1.52-32.15)**	**0.01**
12	C	G	G	C	6 (0.010)	4 (0.004)	2.10 (0.59-7.50)	0.34
13	T	G	G	T	6 (0.010)	8 (0.009)	1.05 (0.36-3.06)	1.00
14	T	T	A	T	0 (0.000)	4 (0.005)	1.72 (1.60-1.84)	0.15
15	C	G	A	T	7 (0.012)	0 (0.000)	**2.34 (2.18-2.63)**	**<0.01**

freq: frequency Ref: reference * Calculated by Fisher’s Exact Test

## Discussion

DNA methylation is the most common epigenetic modification of DNA in mammalian genomes, presenting a heritable epigenetic feature that is associated with transcriptional silencing, X-chromosome inactivation, genetic imprinting, genomic stability, and gene expression. However, aberrant DNA methylation patterns involving hypermethylation or hypomethylation have been associated with the development and progression of various cancers [23].

Previous studies have implicated methylation-specific gene alteration as a major mechanism involved in inappropriate gene activation or silencing in leukemic cells, which has been shown to be a universal feature occurring in all AML patients [24,25]. DNMT3B plays a crucial role in embryonic development and aberrant DNA methylation during carcinogenesis [26]. In addition, polymorphisms in the DNMT3B gene may influence its enzymatic activity, and the SNPs in the DNMT3B promoter that play a role in de novo methylation have also been reported to be associated with several tumor susceptibilities [18-22]. Therefore, the SNPs in DNMT3B may serve as important indicators for genetic susceptibility to AML development, and genetic polymorphism assays are likely to be used for investigating the etiology of AML. However, there are no reports regarding the possible association between AML susceptibility and DNMT3B polymorphisms in Chinese Han populations.

With regard to DNMT3B polymorphisms, previous studies have focused on the transcription start site within the promoter and exon 1B regions, i.e., SNPs rs2424913 and rs1569686, respectively. In our case-controlled study, we identified 5 SNPs in the DNMT3B gene, including rs2424913, rs6087990, rs1569686, rs6119954, and rs2424908, with the first 3 SNPs sites found in promoter regions and the latter 2 in an exon region. We found that the rs2424913 SNP demonstrated no polymorphisms in either the case or control group of Chinese Han individuals, with 100% of this population presenting the TT genotype. This result is consistent with reports from Japan and North China [27,28], with the latter study showing that the C/C genotype in the Chinese population is absent or rare and that the frequency of the C/T genotype is low in the Chinese population (5.1% in the control group) but distinct from that of Caucasians, who have reported frequencies for the T/T, C/T, and C/C genotypes of 23.3, 45, and 31.8% in the British population [19], respectively, and 23.2, 41.8, and 35% in Americans [22], respectively. Because the rs2424913(-149C>T) SNP is located -149 bp from the DNMT3B gene promoter transcription start site, it was reported that a C to T transition induces a 30% increase in promoter activity for the DNMT3B gene [19,22]. Furthermore, genetic polymorphisms often vary across ethnic groups, and the observed diversity in the rs2424913 SNP distribution in different ethnic populations may explain the different methylation status in Chinese and Caucasians populations.

The 2 SNPs in the DNMT3B promoter, rs6087990(-283T > C), which is located -283bp from the exon 1A transcription start site, and rs1569686(-579 G>T), which is located-579bp from the exon 1B transcription start site, are located in CpG-rich and CpG-poor promoters, respectively. We found no significant difference in rs6087990 regarding the genotype distribution and allele frequency between patients with AML and control individuals, although this SNP has been reported to significantly affect promoter activity and was associated with a 50% reduction in lung cancer risk in a Korean population [29]. In addition, our results showed that the frequency of the C allele was higher than that of the T allele in the case and control groups, and the allele frequencies were in accordance with data from the Asian population in the dbSNP public database but in contrast to data from the European population.

Contradictory conclusions have been made regarding the association between rs1569686 and various cancers. Previous studies have demonstrated that the G allele significantly decreased the susceptibility to lung and colon cancer in a Korean population [29,30] and that the GT genotype was associated with a significantly decreased risk for lung cancer (OR: 0.517; 95% CI: 0.273-0.981) compared to the TT genotype in Northern and Southern Chinese populations [31]. However, it was also reported that there was no association between rs1569686 and head and neck squamous cell carcinoma in a Chinese population [32]. Our present study revealed that there was a significant difference in rs1569686 between patients with AML and control individuals in the Chinese Han population; we analyzed the genotype distribution in an additive dominant model and a recessive model, and the results showed that the population with the GG genotype appeared to be 5.76 times more susceptible to AML compared with the population with the TT genotype. Moreover, the allele frequency analysis consistently revealed that the minor G allele was associated with an increased risk for AML compared with the T allele (OR: 1.89; 95%CI: 1.41-2.52; *P<0.01*), thus, rs1569686 can predict the risk of AML in a minority of patients. Previous studies have confirmed that rs1569686 is located in the exon 1B transcription start site region of DNMT3B and that the T transversion affects the transcriptional activity of the DNMT3B gene [33]. Because different variants of DNMT3B may have altered catalytic activity and were expressed in a tissue-specific manner, it was important to explore the genotype distribution of DNMT3B SNPs in different tumor types, particularly those that have produced contradictory results in previous reports.

We found no significant difference in rs6119954 with regards to the genotype distribution and allele frequency between patients with AML and control individuals; however, for rs2424908, which demonstrated strong LD with rs6119954 (D'=0.84), there was a significant association with AML risk. In particular, the CC genotype was associated with a reduced risk for AML compared with the TT genotype (OR: 0.57; 95%CI: 0.36-0.91; *P<0.01*); therefore, the C allele may serve asa protective factor in the development of AML. Similar results were obtained in the subsequent haplotype analysis. For example, the TTGC haplotype, which expresses a C allele in rs2424908, resulted in decreased AML risk compared with the major CTGT haplotype(OR: 0.29; 95%CI: 0.12-0.71; *P<0.01*). In contrast, the haplotypes CGGT, TGAT, and CGAT, which express a G allele in rs1569686 and a T allele in rs2424908, appeared to significantly increase the risk for AML.

To the best of our knowledge, this is the first report to investigate the distribution and association of SNPs in DNMT3B with genetic susceptibility to AML. Our study demonstrated that the G allele in rs1569686 may serve as a risk factor for AML, whereas the C allele in rs2424908 may be a protective factor for the occurrence of AML. Thus, DNMT3B gene polymorphisms might become useful markers for AML epidemiological studies, although confirmation of our findings in larger groups and different populations is required. In future studies, we plan to perform functional analyses of these SNPs, which may lead to a better understanding of the disease mechanisms as well as to the discovery of new biomarkers and/or drug targets and improved rational design for therapeutic regimens.
